# Developing a Personal Leadership Competency Model for Safety Managers: A Systems Thinking Approach

**DOI:** 10.3390/ijerph19042197

**Published:** 2022-02-15

**Authors:** Hassan M. Alidrisi, Sherif Mohamed

**Affiliations:** 1 Department of Industrial Engineering, Faculty of Engineering, King Abdulaziz University, Jeddah 21589, Saudi Arabia; 2 School of Engineering and Built Environment, Griffith University, Brisbane 4222, Australia; s.mohamed@griffith.edu.au

**Keywords:** safety leadership behaviour, leadership model, leadership competencies, systems thinking, complex environment

## Abstract

The roles of all levels of management in influencing safety, particularly in a complex work environment, are crucial. Therefore, safety managers need to develop leadership competencies (i.e., effectiveness in terms of person-oriented behaviours) to reinforce their influencing capabilities through their safety responsibilities. However, practising leadership behaviours without considering how and when these behaviours should be executed is not enough. Therefore, this paper develops a personal leadership competency model by adopting the Systems Thinking approach. The model was developed by conducting exploratory factor analysis and confirmatory factor analysis of three behavioural leadership competencies (emotional, social and cognitive) selected to fulfil the holistic view of Systems Thinking. Data were collected via self-administered questionnaire surveys. A total of 180 valid responses were received from construction managers responsible for overseeing site safety. The statistical results revealed three factors belonging to emotional competency—achievement orientation and adaptability, positive outlook, and emotional self-control. Regarding social competency, four factors represented it—teamwork, organisational awareness, coach and mentor, and conflict management. Finally, cognitive competency was found to be formed by two factors—interaction recognition and pattern recognition. All nine identified factors should, in combination, help safety managers to achieve a better understanding of themselves, of others and of their worksite environments.

## 1. Introduction

As leaders, safety managers play an important role in maintaining the safety of work environments [1]. However, managing safety is a complex process because, according to Berhanu [2], accidents usually happen randomly even though their causes can be predicted. This complexity increases if the work environment is also complex. Managing a complex environment has many challenges, such as dynamic situations that depend on numerous components and containing conflicts between stakeholders. Therefore, safety leaders need to prepare themselves by mastering competencies to better understand themselves, to influence others and to manage the work environment. Regarding the ability to influence others, this paper presents three kinds of leadership competency—emotional, social and cognitive. Aiming to acquire a better understanding of the effect of these three competencies on leaders’ influencing skills, the paper develops a personal leadership competency model that has its roots in the well-known Systems Thinking approach. The model is expected to provide an iterative process for leaders to understand themselves better, others and their work environment.

This paper is structured as follows. The next section briefly introduces the concept of leadership competency(ies), leadership models and categorisations, as reported in the relevant literature. Following this is a presentation of a holistic view of safety leadership, which is explored (as a process) in light of the Systems Thinking approach in order to develop a personal leadership competency model. Section 2 presents the research methodology, data collection and data analysis. Section 3 sheds light on the participants’ profiles as well as the reliability and validity of the measurement scale. Section 4 discusses the findings that emerge from the statistical data analysis and implications for safety leaders. Section 5 describes the study limitations and Section 6 highlights the conclusion.

### 1.1. Leadership Competencies

Boyatzis [3], who came up with one of the earliest definitions of competency in the management context, defined it as ‘the underlying characteristics of a person that lead to, or cause effective and outstanding performance’. The importance of this definition lies in how it originated. According to Boyatzis [4], the basic competency concept originates from job performance theory. This definition clearly aligns with the competencies that leaders need.

Rather than base leadership assessment on personality traits, competency models specify the actions and behaviours needed for successful leaders. In the last two decades, a number of studies developed competency models for safety managers. Blair [5] identified the most needed competencies for safety managers based on a managerial competency model. The findings revealed the most important competencies for the eight different roles of safety managers—communicating effectively (mentor role); obtaining input from others (facilitator role); auditing and analysing the safety effort (monitor role); sharing and exchanging relevant information (coordinator role); translating a solution into practical terms (innovator role); maintaining a positive image and reputation (broker role); accepting responsibility (producer role); and maintaining and sharing a vision for safety (director role).

Based on environmental health and safety (EHS) functions, Leemann [6] developed a model of 19 competencies for safety managers. These competencies are categorised into three clusters—cognitive competencies, interpersonal competencies and intrapersonal competencies. Daud et al. [7] investigated an EHS competency model to identify the most needed competencies for the safety profession. Their main interest was to enhance safety managers’ ability in four tasks (standard setting, enforcement, promotion and specific functions). They classified these competencies into two groups, threshold competencies and differentiating competencies, based on their level of importance.

Instead of developing a competency model for safety managers from an educator’s perspective, Chang et al. [8] introduced a competency model based on the perspectives of safety professionals. They identified the 10 most essential competencies for safety professionals and clustered them into 5 main dimensions: (1) recognising safety and health hazards; (2) measuring, evaluating, and controlling safety and health hazards; (3) safety and health training and management; (4) applying business principles, practices and metrics in safety and health practice; and (5) applying industrial safety and health laws and regulations.

The literature on leadership discusses many different competencies. However, two sets of leadership competencies can encompass all the competencies emphasising the cognitive and emotional functions of leadership [9]. The first set, suggested by Dulewicz and Higgs [10], includes three types of competencies—intellectual, managerial and emotional [9]. The combination of these competencies accounts for 79% of leadership performance [10]. The second set, suggested by Boyatzis et al. [11], includes two competencies, emotional and social, with cognitive competence added later. These two sets are similar in their functions and components, as they emphasise the same functions and have mutual components. Yet, the conceptual basis of the two sets is different. The components of the first set were identified through the functions of leadership [9], whereas the components of the second set were identified through the theory of action and job performance [12].

### 1.2. Safety Leadership and Systems Thinking

Safety leadership, defined as ‘the process of defining the desired state, setting up the team to succeed, and engaging in the discretionary efforts that drive the safety value’ [13], is widely recognised to be critical [14]. However, the role of safety leadership is changing and is becoming multi-dimensional. On the one hand, safety leadership requires a rigorous understanding of the systems that control hazards and reduce exposure. On the other hand, it is also more personal. Leaders who know themselves and understand their effects on their relationships, teams and organisational culture enable themselves to be more effective [15]. It is in this context that the authors argue that contemporary safety leadership should be viewed as a complex process through the lens of Systems Thinking, as described below.

Systems Thinking offers an interdependent view of systems [16], as it assumes that one event in the system could impact the other. This allows the understanding of the linear and non-linear cause-and-effect relationships, in addition to the underlying pattern of events [17]. It also adopts a dynamic view as a way of making sense of the context [16]. On this basis, Systems Thinking and the holistic view it offers represent an ideal approach to better understand leadership competencies considering the leader, followers and work environment and how all these interact with each other. To facilitate this understanding, this study argues that Systems Thinking—which gives an interdependent view of systems [18]—has the ability to provide a leadership competency model that focuses on the elements of the influencing process of leadership.

The aforementioned competency models focus on the management role of safety professionals. The lack of a personal leadership competency model concerned with leaders’ safety responsibility should be noted, and its importance lies in the essential role of leadership in influencing safety [19]. Moreover, whereas the previous studies identified and then categorised the competencies based on responsibilities without investigating the leadership itself as a process, the competencies developed in this study are part of a bigger model that reflects the process of leadership. The following section sheds light on the developed competencies as part of the whole leadership process, unlike previous studies that have neglected the process.

### 1.3. Safety Leadership as a Process

To comprehend the whole of a system, Gharajedaghi [20] suggests applying an iterative process based on four independent variables comprising a relationship cycle. These variables are (1) function, (2) structure, (3) process, and (4) context. Each variable works as a co-producer for other variables, and the cycle is closed once the holistic view is achieved. Gharajedaghi [20] also discusses the difficulty of seeing the whole in the case of failing to figure out these interdependencies. Therefore, the iterative process is of great importance in understanding the complexity of a whole system.

**Figure 1:**
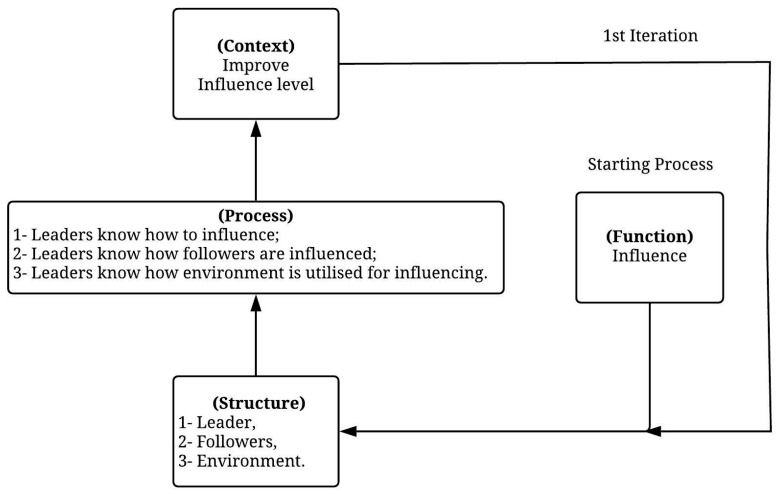
Iterative process for understanding leadership.

In this paper, applying the iterative process to the adopted definition of leadership can help to comprehend and interpret the leadership concept holistically (Figure 1). The definition of leadership used in the study is that developed by Northouse [21]; leadership is “a process whereby an individual influences a group of individuals to achieve a common goal”. The leader, the followers and the environment make up the structure of leadership. The leaders’ ability to influence followers, their ability to understand how their followers are influenced, and their ability to understand the environment to allow efficient influencing comprise the process explaining the manner in which the structure generates leadership functions. By developing an understanding of the relationship between the function, the structure and the process and placing leadership in a suitable context afterwards, the ability to achieve the desired leadership-driven targets is possible. Therefore, it can be said that such a holistic view can provide a helpful explanation in the midst of understanding how leaders influence their followers.

The holistic view of safety leadership may be explained as the manner in which leaders utilise their leadership abilities, their followers’ abilities, and the environment. Knowing how to influence, how followers are influenced and how to use the environment for influencing is essential for leaders to effectively practice safety leadership. Both Mumford et al. [22] and Yukl and Mahsud [23] have proposed a view of flexible leadership that aligns well with the proposed holistic view. Mumford et al. [22] contend there is more to leadership than just practising influencing behaviour. Yukl and Mahsud [23] state that, to provide leaders with a better understanding of themselves, others and the environment, emotional, social and cognitive competencies are required. By reinforcing these three competencies, leaders become more competent in influencing their followers and are provided with flexibility and a holistic view when exercising their leadership roles.

Many studies, such as Palaima and Skaržauskiene [16], Goleman [24] and Boyatzis [4], contend that adopting these competencies could lead to outstanding performances in leadership. Boyatzis [4] defines the above-mentioned three competencies as follows:

- Emotional competency is ‘the ability to recognise, understand, and use emotional information about oneself that leads to or causes effective or superior performance’.
- Social competency is ‘the ability to recognise, understand and use emotional information about others that leads to or causes effective or superior performance’.
- Cognitive competency is ‘the ability to think [about] or analyse information and situations that leads to or causes effective or superior performance’.

It has been found that people with a higher emotional competency experience outstanding success in their lives; therefore, there is a relationship between emotional competency, which includes social competency, and positive social behaviour [25,26]. According to Boyatzis et al. [11], their Emotional Competence Inventory 360 (ECI 360) model is related to an individual’s performance on a work site. Bar-On [27] is another leading research study exploring emotional competency. The study developed an emotional competency model that increases individuals’ ability to deal with their work environments [25]. The researchers who established these models contend that the combination of emotional competency (and social competency) and cognitive competency provides a greater chance of better performance [4,24,28].

### 1.4. Complex Work Environment

Many industries, such as the aviation, oil and gas and construction industries, have complex work environments. The current study selected the construction industry as its applied setting for the following reasons: (1) the industry has one of the worst workplace accident records worldwide; (2) construction site management perceive projects as complex, dynamic phenomena in a non-linear setting; (3) a typical construction site is a work environment where humans are expected to interact, but because of its temporary character, the site has a highly transient social system; and (4) construction projects are dynamic, as virtually all supplies and resources are highly dependent on the world surrounding the project. Therefore, this study uses the construction industry as the setting to develop a personal leadership competency model for construction safety leaders.

## 2. Research Methodology

### 2.1. Population and Sample

The investigation area of this study is the construction industry in Saudi Arabia. According to the objective of the study, safety leaders—such as project managers, safety managers or any other managers with safety responsibilities—were considered the target population. In total, more than 500 questionnaires were collected from the participants by snowball sampling method. A large number of returned responses were incomplete. This resulted in data analysis using 180 completed questionnaires.

### 2.2. Measures

The Emotional and Social Competency Inventory (ESCI-U) instrument established by Boyatzis and Goleman [29] was adopted and adjusted for the context of construction safety. It comprises three sections (emotional, social and cognitive competency). The details of each section are as follows:

- The **emotional competency** section includes 21 items, originally distributed into 5 factors: (1) emotional self-awareness (factor ESA, 4 items); (2) achievement orientation (factor AO, 4 items); (3) adaptability (factor A, 4 items); (4) emotional self-control (factor ESC, 4 items); and (5) positive outlook (factor PO, 5 items). The first factor belongs to the self-awareness cluster, whereas the remaining four factors are associated with the self-management cluster.
- The **social competency** section includes 28 items, originally distributed into 7 factors: (1) empathy (factor E, 4 items); (2) organisational awareness (factor OA, 4 items); (3) conflict management (factor C, 4 items); (4) coach and mentor (factor CM, 4 items); (5) influence (factor I, 4 items); (6) inspirational leadership (factor IL, 4 items); and (7) teamwork (factor T, 4 items). The first two factors belong to the social awareness cluster, whereas the remaining five are associated with the relationship management cluster.
- The **cognitive competency** section includes 10 items, originally distributed into 2 factors: (1) interaction recognition (factor IR, 5 items) and (2) pattern recognition (factor PR, 5 items).

Finally, it is important to mention that a five-point Likert-type scale was employed to measure leaders’ awareness of the items, where 1 = never, 2 = rarely, 3 = sometimes, 4 = often and 5 = always.

### 2.3. Data Analysis

A series of quantitative approaches were applied in the presented study, specifically descriptive analyses, exploratory factor analysis (EFA) and confirmatory factor analysis (CFA). The descriptive analyses were employed to check the data reliability using statistical techniques, such as Cronbach’s alpha analysis. Assessing the validity of the measurement scale was performed using EFA and CFA, sequentially. This was accomplished using the statistical package for social science (SPSS 24.0, IBM Corp., Armonk, NY, USA) and AMOS 25.0 (IBM Corp., Armonk, NY, USA), an extension of SPSS.

## 3. Results

### 3.1. Overview of the Participants

Almost half of the respondents were employed as construction site managers (49.7%), whereas 32.7% and 17.6% worked in project manager and safety manager positions, respectively. Regarding work experience, more than 40% had more than 5 years of experience. Regarding the organisation safety performance level, 40.3% of the participants believed they were working in organisations with safety performance levels the same as the average level of the local industry, whereas 35.2% believed safety levels in their organisation were below average and 24.5% believed safety levels were above average.

According to the analysis, the responses were considered a suitable representation of the opinions of the population for two reasons. First, the majority of the participants had notable experiences. Second, the participants reflected a good mix of organisation safety performance levels.

### 3.2. Validity and Reliability of the Safety Competency Scale

#### 3.2.1. Item Analysis

Based on the five-point Likert-type scale, the mean values of the emotional competency variables seem to be high. All mean values were above 3.75, ranging from 3.79 to 4.45. More specifically, the professional individuals were perceived as seeking to do things in a safer way and as hard workers who improved their safety performance, as indicated by the two highest mean values EI2BQ3 (4.45, SD = 0.691) and EI2BQ4 (4.30, SD = 0.739); see Table A1 for item descriptions. Interestingly, despite their interest in adopting safety behaviours, such as in variable EI2BQ3, their planning and strategy to cope with unexpected safety accidents had the lowest score (EI2CQ3: 3.79, SD = 0865). Evidently, showing the ability to deal with stress in unsafe situations was not the main attribute of these professionals when compared with other perceived characteristics, such as variable EI1AQ1 (showing awareness of their own feelings regarding safety concerns; 4.27, SD = 0.780) and variable EI2CQ1 (applying safety standard procedures flexibly; 4.20, SD = 0.759). These variables were more obvious, particularly as emotional competency mainly depends on understanding and utilising one’s feelings and abilities. A description of each emotional competency item along with its mean and standard deviation values is presented in Table A1.

Except for one variable, all other mean values of social competency variables were above the mean level of 3.00. The mean values ranging from 3.58 to 4.36 and a single variable (SI2JQ2: convincing others by appealing to their self-interest; 2.91, SD = 1.373) were significantly below the range. The participants believed strongly in being respectful and supportive of other team members, as indicated by the two highest mean values SI2LQ1 (4.36, SD = 0.713) and SI2LQ2 (4.32, SD = 0.680). Interestingly, in addition to the two highest variables, two other variables, SI2LQ3 (4.13, SD = 0.766) and SI2LQ4 (4.02, SD = 0.776), more related to building team capability and working co-operatively, were also significantly higher compared to other variables. Evidently, understanding the reasons for unsafe actions taken by others was not the main aspect for the participants (SI1FQ3; 3.58, SD = 1.077), despite their strong belief in understanding others’ concerns about safety and understanding others from different backgrounds, SI1FQ1 (4.29, SD = 0.705) and SI1FQ2 (4.02, SD = 0.796). A description of each social competency item along with its mean and standard deviation values is presented in Table A2.

With a similar outcome, the overall levels of cognitive competency variables were perceived to be strong. All the variables had mean values greater than 3.50, which means the respondents had a strong belief in the characteristics of their cognitive competency. The highest mean value was 4.11 (SD = 0.758), which was concerned with how safety accidents are viewed as a cause–effect relationship (CIMQ5). In addition, the participants were perceived to consider safety when they explained complex processes. In contrast, interpreting a new situation using a story relating it to a different type of situation had the lowest mean value (CINQ5; 3.77, SD = 0.948). A description of each cognitive competency item along with its mean and standard deviation values is presented in Table A3.

#### 3.2.2. Factorability of Data

The factorability of data was tested using the Kaiser–Meyer–Olkin (KMO) test and Bartlett’s test of sphericity. Table 1 shows that all three constructs (emotional competency, social competency, and cognitive competency) had KMO values greater than 0.60, which is the minimum agreement level acceptable [30]. The values ranged from 0.891 to 0.908. Therefore, these results were good and indicated sampling adequacy. Regarding Bartlett’s test of sphericity, all the statistical values were significant at *p* < 0.001. Therefore, there were adequate relationships between the variables [31]. These results thus confirmed the factorability of each construct for conducting the EFA.

#### 3.2.3. EFA Results

**Figure 2:**
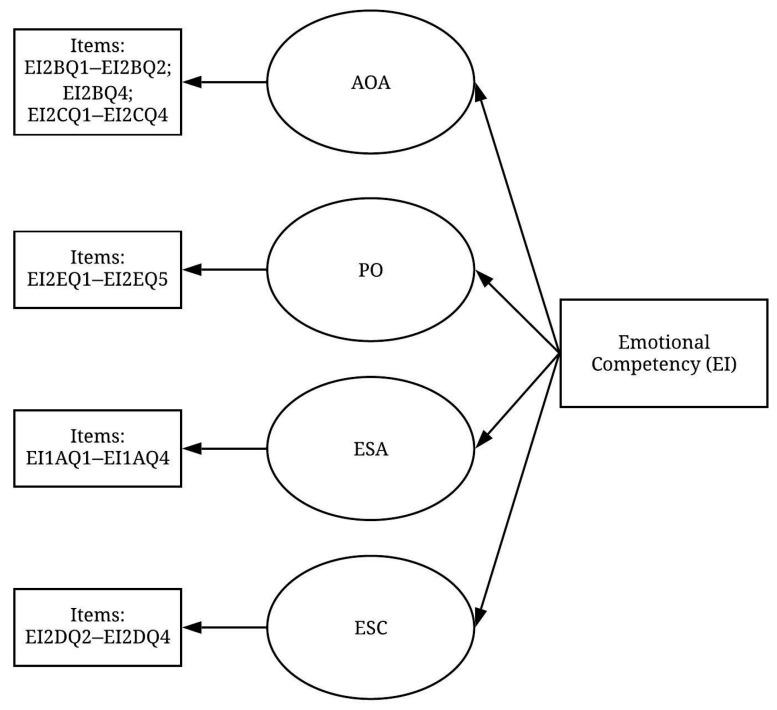
Pre-measurement model of the EI construct (items displayed in Table A1).

Principal component analysis and the Varimax orthogonal rotation method were applied to the three constructs to perform factor extraction and rotation. The scree test, which is used with the principal component analysis, was applied to determine the number of factors that should be retained [32]. This test recognised four sub-factors, which explained 64.7% of the total variance for the emotional competency (EI) construct. Table 2 shows the patterns of the rotated component matrix that indicate most of the variables were found to significantly exceed the threshold level of 0.5. One variable was deleted due to its failure to reach the acceptable level (EI2BQ3; 0.478). Another variable, EI2DQ1 (I act safely even in emotionally charged situations), was deleted because, theoretically, it does not belong to the extracted factor. As a result, 4 factors were identified from the remaining 19 variables, and a pre-measurement model of the EI construct was developed (Figure 2).

**Figure 3:**
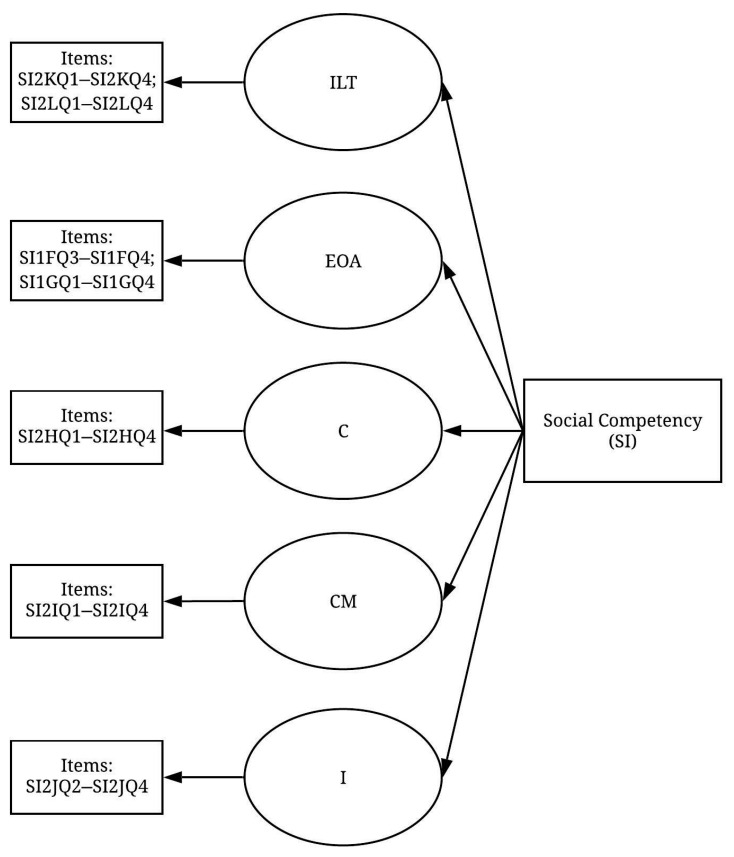
Pre-measurement model of the SI construct (items displayed in Table A2).

As for the social competency (SI) construct, both the scree test and the eigenvalue suggested that five factors should be derived from the SI construct. These five factors explained 62.96% of the total variance. Table 3 shows the patterns of the rotated component matrix indicating that most of the variables were found to significantly exceed the threshold level of 0.5. One variable was deleted due to its failure to reach the acceptable level (SI1FQ2; 0.491). Two more variables, EI2FQ1 (I understand others’ concerns about safety by listening attentively) and SI2JQ1 (In safety matters, I convince others by getting support from key people), were deleted because, theoretically, they do not belong to the extracted factor. Therefore, 5 factors were derived from the remaining 24 variables, and a pre-measurement model of the SI construct was developed (Figure 3).

**Figure 4:**
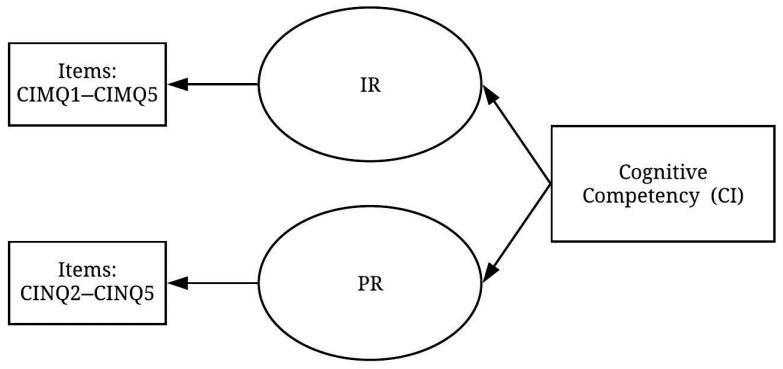
Pre-measurement model of the CI construct (items displayed in Table A3).

As for the cognitive competency (CI) construct, the scree test recognised two sub-factors that explained 65.54% of the total variance for the CI construct. Table 4 shows the patterns of the rotated component matrix that indicate all the variables were found to significantly exceed the threshold level of 0.5. However, one variable CINQ1 (I perceive similarities among different types of situations) was deleted due to cross-loading. As a result, two factors were identified from the remaining nine variables, and a pre-measurement model of the CI construct was developed (Figure 4).

#### 3.2.4. CFA Results

**Figure 5:**
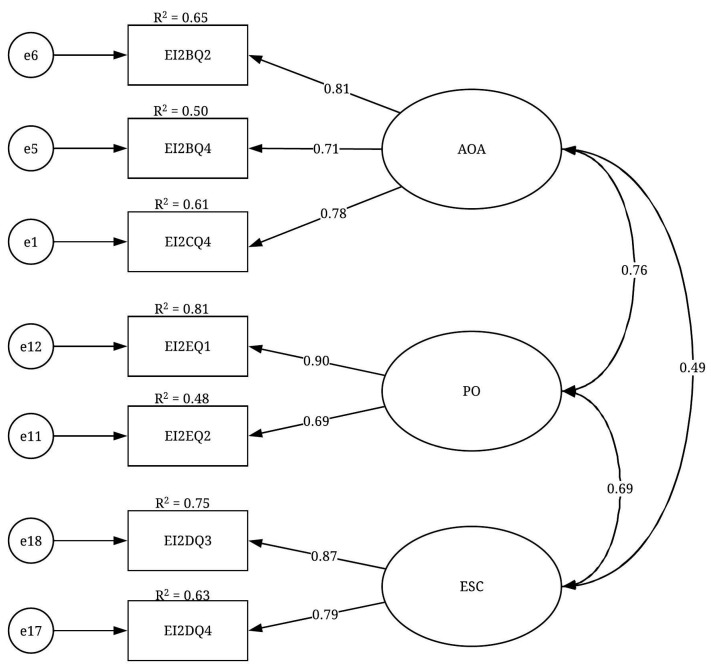
CFA model of the EI construct.

The CFA results of the EI construct are presented in Table 5. The CFA results suggested deleting the ESA factor and keeping the remaining three factors of achievement orientation and adaptability (AOA), ESC and PO. All the fit indices of the EI construct indicated that the final CFA model of this construct (Figure 5) had a good fit level: X^2^/df = 1.707; GFI = 0.972; IFI = 0.986; TLI = 0.973; CFI = 0.986 and RMSEA = 0.063. Moreover, the factor loadings were significant at the *p* < 0.001 level, ranging from 0.691 to 0.901. These results were considered relatively high and suggested convergent validity. Regarding the variables’ reliability, almost all the R^2^ values were greater than 0.50, indicating a good level of reliability. Regarding the discriminant validity of the construct, all correlation coefficients between each pair of factors were less than 0.850, which confirmed the discriminant validity of the EI construct. Finally, as the acceptable level of the fit indices was achieved, the unidimensionality for this construct was confirmed.

**Figure 6:**
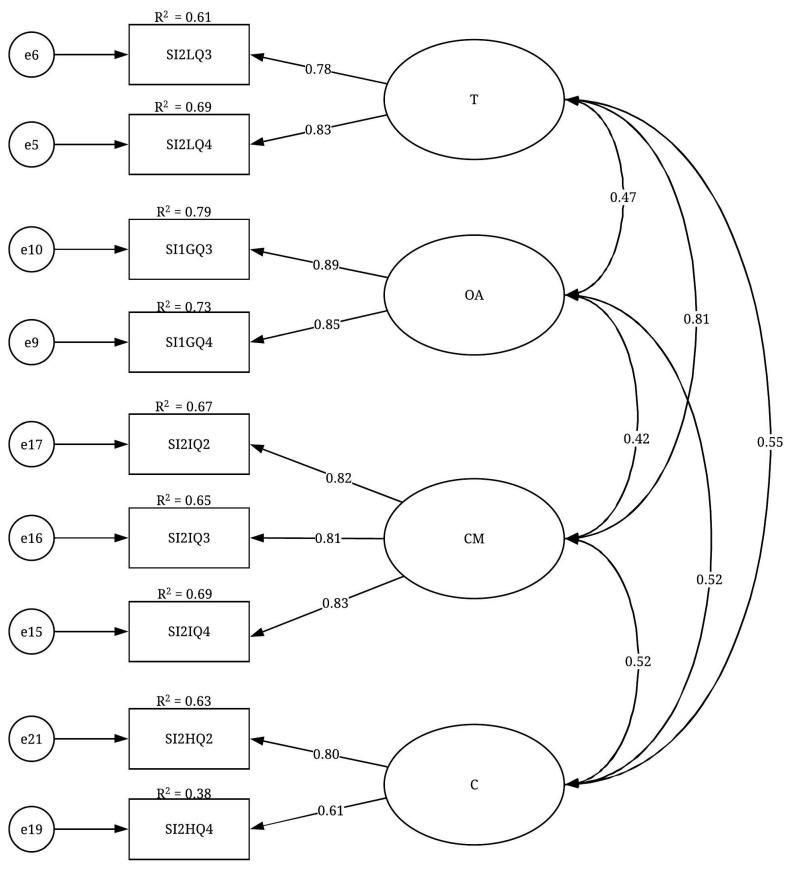
CFA model of the SI construct.

The CFA results of the SI construct are presented in Table 6. The CFA results suggested deleting the influence (I) factor and differentiated and keeping the T factor from the ILT factor and the OA factor from the EOA factor. The results also suggested keeping the remaining two factors, CM and C. All the fit indices of the SI construct indicated that the final CFA model of this construct (Figure 6) had a good fit level: X^2^/df = 1.517; GFI = 0.962; IFI = 0.985; TLI = 0.975; CFI = 0.985 and RMSEA = 0.054. Moreover, the factor loadings were significant at the *p* < 0.001 level, ranging from 0.613 to 0.888. These results were considered relatively high and suggested convergent validity. Regarding the variables’ reliability, almost all the R^2^ values were greater than 0.50, indicating a good level of reliability. Regarding the discriminant validity of the construct, all the correlation coefficients between each pair of factors were less than 0.850, which confirmed the discriminant validity of the SI construct. Finally, as an acceptable level of the fit indices was achieved, the unidimensionality for this construct was confirmed.

**Figure 7:**
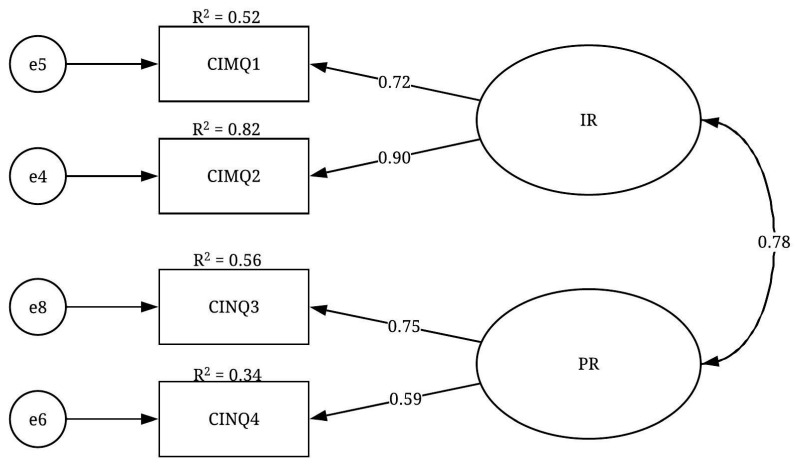
CFA model of the CI construct.

The CFA results of the CI construct are presented in Table 7. The CFA results suggested two factors, IR and PR. All the fit indices of the CI construct indicated that the final CFA model of this construct (Figure 7) had a good fit level: X^2^/df = 0.023; GFI = 1.0; IFI = 1.0; TLI = 1.0; CFI = 1.0 and RMSEA = 0.000. Moreover, the factor loadings were significant at the *p* < 0.001 level, ranging from 0.587 to 0.904. This result was considered relatively high and suggested convergent validity. Regarding the variables’ reliability, almost all the R^2^ values were greater than 0.50, indicating a good level of reliability. Regarding the discriminant validity of the construct, the correlation coefficient between the two factors was less than 0.850, which confirmed the discriminant validity of the CI construct. Finally, as an acceptable level of the fit indices was achieved, the unidimensionality for this construct was confirmed. In summary, the CFA results revealed three, four and two factors belonging to the emotional, social and cognitive constructs, respectively. The listed items for these factors are presented in Table 8.

## 4. Discussion

**Figure 8:**
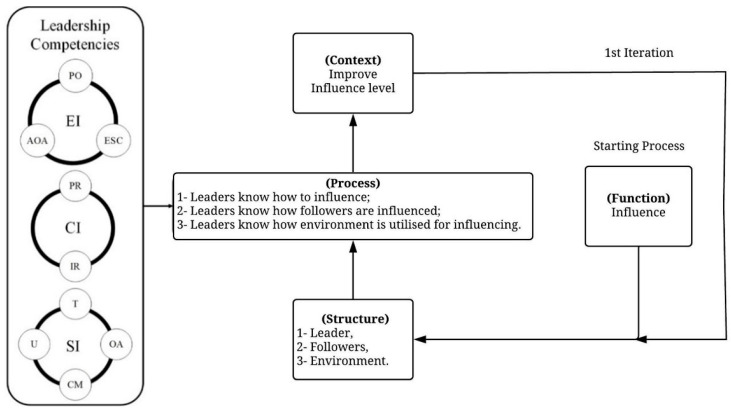
The role of leadership competencies in understanding the leadership influencing process.

The objective of this study was to develop a personal leadership competency model for safety leaders and to investigate the leadership competencies needed to successfully fulfil the influencing process demonstrated by the Systems Thinking concept. The three selected leadership competencies (emotional, social and cognitive) were proven to be a part of the model. These results are compatible with Boyatzis [4] and Yukl and Mahsud [22], as they emphasised the role these competencies have in leaders’ influencing process. Based on the findings, safety leaders need the three competencies to enhance their ability to understand the leadership influencing process that involves leaders, followers and the environment (Figure 8).

For emotional competency, the CFA results revealed the importance of three competency factors: (1) Achievement Orientation and Adaptability (AOA); (2) Positive Outlook (PO); and (3) Emotional Self-Control (ESC). The AOA factor helps safety leaders to achieve a standard of excellence in their role, which requires flexibility, such as changing perceptions and ideas based on new input. This could be mastered by safety leaders by regularly consulting others and reviewing the way their work is performed in order to assess safety situations and by investing more time to recall previous unsafe situations to evaluate their responses to those situations. Regarding the PO factor, it helps safety leaders to look at difficult situations as opportunities for learning and improvement. This could be mastered by taking notes about responses to situations and how to turn them into learning and improvement opportunities. The ESC factor helps safety leaders to develop the ability to keep negative actions under control when provoked. This could be mastered by understanding the implications of their safety actions and controlling the potential triggers of losing self-control. Collectively, these three competencies provide safety leaders with the ability to enhance their safety performance. 

Regarding social competency, the CFA results revealed the importance of four competency factors: (1) Teamwork (T); (2) Organisational Awareness (OA); (3) Coach and Mentor (CM); and (4) Conflict Management (C). The T factor supports safety leaders’ ability to discuss and share safety matters with their followers. This could be mastered by regularly asking followers to share their opinions regarding safety aspects. Regarding the OA factor, it helps safety leaders to understand their organisations’ culture and identify those with the influencing power in their teams by understanding the cultural norms of their organisations. The CM factor is concerned with improving safety leaders’ ability to set up a continuous development program for team members. This competency is achieved by providing followers with constructive feedback and demonstrating what they could do differently, how they could improve their safety and pointing out their strengths that can be utilised to improve their safety performance. Moreover, leaders may take time for a friendly chat about things they may want feedback on. Regarding the C factor, it helps safety leaders to focus on the issues of conflict and work on de-escalating the associated negative feelings. This could be mastered by openly discussing the views on safety that are disagreed upon before conflict arises. Collectively, these four competencies provide safety leaders the ability to enhance their followers’ safety performance.

Regarding cognitive competency, the CFA results revealed the importance of two competency factors: (1) Interaction Recognition (IR) and (2) Pattern Recognition (PR). The IR factor helps safety leaders to identify and view an event as cause and effect. This competency could be mastered by spending time to recall previous unsafe situations to analyse and connect the people or other events that could have caused and affected the situation. Regarding the PR factor, it helps safety leaders to recognise and assess safety situation patterns and see the commonality among diff0erent safety situations. Collectively, these two competencies provide safety leaders with the ability to prepare worksite environments for better safety performance.

As leaders execute safety practices, the how and when of these practices must be considered. The Systems Thinking approach considers these two aspects in terms of behaviour in complex environments. This is fundamental in improving leaders’ influencing process, which can be achieved through the nine identified competencies. Competencies IR and PR deal with understanding the environment, whereas competencies AOA, PO and ESC are about understanding the leaders themselves. At the beginning, novice safety leaders might attempt to assess and build an understanding of how elements of a worksite environment impact each other. As they learn to prepare worksite environments, they are strongly advised to start the iteration 1 where competencies AOA, PO and ESC help them to understand themselves. This will help to improve their safety level at their worksites. As they improve this skill, they may opt for the next iteration where competencies T, OA, CM and C are related to their relationship with their followers. These competencies aim to understand followers’ perceptions and help to improve their safety performance, aligning it with the targeted safety level.

From the above, and to improve influencing skills, it could be recommended that safety leaders need to:

- View a safety event as cause and effect.
- Assess safety situation patterns regularly.
- Consult others regarding safety situations.
- Record and review safety situational responses.
- Improve self-discipline.

To further enhance safety leaders’ influencing skills, they are encouraged to:

- Share their safety opinions among their team.
- Learn the cultural norms of their organisations.
- Set up a continuous development program for their followers.
- Discuss divergent views before conflict arises.

## 5. Study Limitations

Two potential limitations were noted about the current research study. First, the main concern of this study was to develop a personal leadership competency for safety managers who work in complex environments. All the voluntary participants were from the construction industry. Although the environment of this industry is considered one of the most complex environments, different environments could have revealed different competency models. Therefore, a sample from other industries with complex environments is suggested to generalise this model for complex environments. Second, similar to other research studies that use self-assessment surveys to collect data, the findings of this study may have been exposed to some amount of bias. Despite both limitations, this study offers support for the holistic competency model for safety managers.

## 6. Conclusions

This study aimed to develop a personal leadership competency model for safety managers in a complex environment with the help of the concept of Systems Thinking. The study identified nine leadership competencies categorised into three main domains—emotional, social and cognitive. Collectively, these competencies enable safety leaders to understand the complexities that face them in their work environment. This research study has opened up more questions that require exploration. Further investigation of the effect of this leadership competency model on safety outcomes would be useful. This would test the impact of these competencies on safety performance in complex environments.

## Figures and Tables

**Table 1:** Kaiser–Meyer–Olkin test and Bartlett’s test of sphericity.

Construct	KMO	Bartlett’s Test of Sphericity		
		Approx. Chi-Square	df	Sig.
Emotional competency	0.908	1809.838	210	0.000
Social competency	0.891	2457.044	378	0.000
Cognitive competency	0.901	824.622	45	0.000

**Table 2:** Rotated factor loading of the IE construct.

Variable	Rotated Component			
	1	2	3	4
EI2CQ3	0.742	Achievement Orientation and Adaptability		
EI2BQ2	0.723			
EI2CQ4	0.693			
EI2CQ1	0.686			
EI2DQ1 *	0.657			
EI2BQ1	0.654			
EI2CQ2	0.653			
EI2BQ4	0.594			
EI2EQ3		0.745	Positive Outlook	
EI2EQ2		0.719		
EI2EQ5		0.699		
EI2EQ4		0.671		
EI2EQ1		0.575		
EI2BQ3 *				
EI1AQ3		Emotional Self-Awareness	0.848	
EI1AQ2			0.807	
EI1AQ4			0.724	
EI1AQ1			0.533	
EI2DQ3		Emotion-al Self-Control		0.803
EI2DQ4				0.789
EI2DQ2				0.711

* Variable was deleted.

**Table 3:** Rotated factor leading of the SI construct.

Variable	Rotated Component				
	1	2	3	4	5
SI2LQ1	0.762	Inspirational Leadership & Teamwork			
SI2LQ2	0.746				
SI2KQ2	0.743				
SI2KQ4	0.708				
SI2LQ4	0.658				
SI2KQ3	0.656				
SI1FQ1 *	0.625				
SI2KQ1	0.620				
SI2LQ3	0.616				
SI1GQ4		0.801	Empathy & Organisational Awareness		
SI1GQ3		0.784			
SI1FQ3		0.716			
SI1GQ2		0.654			
SI1FQ4		0.603			
SI1GQ1		0.544			
SI1FQ2 *					
SI2IQ4			0.771	Coach and Mentor	
SI2IQ3			0.760		
SI2IQ2			0.664		
SI2JQ1 *			0.596		
SI2IQ1			0.541		
SI2HQ4			Conflict Management	0.762	
SI2HQ3				0.755	
SI2HQ1				0.669	
SI2HQ2				0.633	
SI2JQ3				Influence	0.779
SI2JQ2					0.673
SI2JQ4					0.593

* Variable was deleted.

**Table 4:** Rotated factor loading of the CI construct.

Variable	Rotated Component	
	1	2
CIMQ2	0.805	Interaction Recognition
CIMQ1	0.775	
CIMQ4	0.744	
CIMQ3	0.731	
CIMQ5	0.691	
CINQ1 *	0.551	0.520
CINQ5	Pattern Recognition	0.888
CINQ4		0.828
CINQ3		0.606
CINQ2		0.566

* Variable was deleted.

**Table 5:** CFA results of the EI construct.

Factor/Variable	Factor Loading	*t*-Value	R²	Correlations between Factors
AOA: Achievement Orientation and Adaptability				AOA-PO: 0.76
EI2BQ2: I initiate safety actions to improve our work environment.	0.808	10.056	0.653	AOA-ESC: 0.49
EI2BQ4: I strive to improve my own safety performance.	0.709	9.019	0.502	PO-ESC: 0.69
EI2CQ4: I consider safety when I shift priorities and experience rapid change.	0.781	f.p. *	0.61	
PO: Positive Outlook				
EI2EQ1: I see safety rules as a work enabler rather than a work constraint.	0.901	9.166	0.812	
EI2EQ2: I see the positive side in people expressing their safety concerns more often than the negative side.	0.691	f.p. *	0.478	
ESC: Emotional Self-Control				
EI2DQ3: I control my impulses appropriately in unsafe situations.	0.868	8.934	0.753	
EI2DQ4: I remain calm in stressful unsafe situations.	0.794	f.p. *	0.631	

* Fixed parameter for estimation.

**Table 6:** CFA results of the SI construct.

Factor/Variable	Factor Loading	*t*-Value	R²	Correlations between Factors
T: Teamwork				T-OA: 0.47
SI2LQ3: I work well in teams by soliciting others’ input regarding safety.	0.779	9.997	0.608	T-CM: 0.81
SI2LQ4: I work well in teams by encouraging cooperation in safety matters.	0.832	f.p. *	0.692	T-C: 0.55
OA: Organizational Awareness				OA-CM: 0.42
SI1GQ3: I understand the informal processes by which work is achieved in the team or organisation.	0.888	8.612	0.789	OA-C: 0.52
SI1GQ4: I understand the informal structure in the team or organisation.	0.854	f.p. *	0.729	CM-C: 0.52
CM: Coach and Mentor				
SI2IQ2: I coach and mentor others about safety.	0.818	11.96	0.669	
SI2IQ3: I personally invest time and effort in developing others’ safety performance.	0.807	11.788	0.651	
SI2IQ4: I provide on-going safety mentoring.	0.831	f.p. *	0.69	
C: Conflict Management				
SI2HQ2: To avoid unsafe situations, I try to resolve conflict by openly talking about disagreements with those involved.	0.795	5.392	0.632	
SI2HQ4: When resolving conflict, I de-escalate the emotions in the situation.	0.613	f.p.*	0.375	

* Fixed parameter for estimation.

**Table 7:** CFA results of the CI construct.

Factor/Variable	Factor Loading	*t*-Value	R²	Correlations between Factors
IR: Interaction Recognition				IR-PR: 0.78
CIMQ1: I see a situation as multiple cause and effect interactions impacting safety.	0.718	7.717	0.516	
CIMQ2: I explain how certain things affect others resulting in a particular outcome that may affect safety.	0.904	f.p. *	0.817	
PR: Pattern Recognition				
CINQ3: I perceive common trends in work accidents.	0.747	5.824	0.558	
CINQ5: I interpret a new situation by using a story relating it to a different type of situation.	0.587	f.p. *	0.345	

* Fixed parameter for estimation.

**Table 8:** Summary of the CFA results.

Construct	Factor	Item: Description
Emotional Competency (IE)	Achievement Orientation and Adaptability (AOA)	EI2BQ2: I initiate safety actions to improve our work environment.
		EI2BQ4: I strive to improve my own safety performance.
		EI2CQ4: I consider safety when I shift priorities and experience rapid change.
	Positive Outlook (PO)	EI2EQ1: I see safety rules as a work enabler rather than a work constraint.
		EI2EQ2: I see the positive side in people expressing their safety concerns more often than the negative side.
	Emotional Self-Control (ESC)	EI2DQ3: I control my impulses appropriately in unsafe situations.
		EI2DQ4: I remain calm in stressful unsafe situations.
Social Competency (SI)	Teamwork (T)	SI2LQ3: I work well in teams by soliciting others’ input regarding safety.
		SI2LQ4: I work well in teams by encouraging cooperation in safety matters.
	Organisational Awareness (OA)	SI1GQ3: I understand the informal processes by which work is achieved in the team or organisation.
		SI1GQ4: I understand the informal structure in the team or organisation.
	Coach and Mentor (CM)	SI2IQ2: I coach and mentor others about safety.
		SI2IQ3: I personally invest time and effort in developing others’ safety performance.
		SI2IQ4: I provide on-going safety mentoring.
	Conflict Management (C)	SI2HQ2: To avoid unsafe situations, I try to resolve conflict by openly talking about disagreements with those involved.
		SI2HQ4: When resolving conflict, I de-escalate the emotions in the situation.
Cognitive Competency (CI)	Interaction Recognition (IR)	CIMQ1: I see a situation as multiple cause and effect interactions impacting safety.
		CIMQ2: I explain how certain things affect others resulting in a particular outcome that may affect safety.
	Pattern Recognition (PR)	CINQ3: I perceive common trends in work accidents.
		CINQ5: I interpret a new situation by using a story relating it to a different type of situation.

## Data Availability

The data that support the findings of this study are available on request from the corresponding author (H.M.A.). The data are not publicly available due to privacy and ethical restrictions.

## Appendix A

**Table A1:** Descriptive items and statistical analysis of emotional competency (EI) variables.

Variable: Description	Mean	Std. Deviation
EI1AQ1: I show awareness of my own feelings regarding safety concerns.	4.27	0.780
EI1AQ2: I acknowledge my own strengths and weaknesses within the safety context.	4.05	0.907
EI1AQ3: I am able to describe how my feelings affect my safety actions.	3.89	0.816
EI1AQ4: I understand the connection between upcoming safety issues and my own feelings.	3.93	0.793
EI2BQ1: I seek to improve safety conditions by setting higher goals.	4.10	0.864
EI2BQ2: I initiate safety actions to improve our work environment.	4.06	0.854
EI2BQ3: I seek ways to do things in a safer manner.	4.45	0.691
EI2BQ4: I strive to improve my own safety performance.	4.30	0.739
EI2CQ1: I apply safety standard procedures flexibly.	4.20	0.759
EI2CQ2: I smoothly juggle multiple demands in safety-related accidents.	4.12	0.820
EI2CQ3: I plan suitable overall strategy, goals or projects to cope with unexpected safety-related accidents.	3.79	0.865
EI2CQ4: I consider safety when I shift priorities and experience rapid change.	4.07	0.821
EI2DQ1: I act safely, even in emotionally charged situations.	4.00	0.838
EI2DQ2: I remain composed, even in unsafe situations.	3.94	0.889
EI2DQ3: I control my impulses appropriately in unsafe situations.	3.85	0.805
EI2DQ4: I remain calm in stressful unsafe situations.	3.80	0.880
EI2EQ1: I see safety rules as a work enabler rather than a work constraint.	4.12	0.811
EI2EQ2: I see the positive side in people expressing their safety concerns more often than the negative side.	4.01	0.805
EI2EQ3: I see learning opportunities in safety incidents rather than punishing or blaming.	4.17	0.802
EI2EQ4: I am optimistic when thinking about future safety performance.	4.23	0.747
EI2EQ5: I believe our safety record in the future will be better than the previous one(s).	4.24	0.743

**Table A2:** Descriptive items and statistical analysis of social competency (SI) variables.

Variable: Description	Mean	Std. Deviation
SI1FQ1: I understand others’ concerns about safety by listening attentively.	4.29	0.705
SI1FQ2: I understand others from different backgrounds who are concerned or not concerned about safety.	4.02	0.796
SI1FQ3: I understand the reasons for someone else’s unsafe actions at work.	3.58	1.077
SI1FQ4: I understand others’ perceptions of safety rules when they are different from my own.	3.77	0.855
SI1GQ1: I understand the importance of social networks in improving our safety performance.	4.16	0.890
SI1GQ2: I understand the team’s or organisation’s unspoken rules that might affect safety.	3.92	0.883
SI1GQ3: I understand the informal processes by which work is achieved in the team or organization.	3.97	0.882
SI1GQ4: I understand the informal structure in the team or organization.	3.78	0.944
SI2HQ1: To avoid unsafe situations, I try to resolve conflicts by finding a solution that addresses everyone’s interests.	4.02	0.878
SI2HQ2: To avoid unsafe situations, I try to resolve conflict by openly talking about disagreements with those involved.	4.10	0.799
SI2HQ3: To avoid unsafe situations, I try to resolve conflict by finding a common ground position everyone involved can endorse.	4.00	0.847
SI2HQ4: When resolving conflict, I de-escalate the emotions in the situation.	4.13	0.814
SI2IQ1: I provide feedback that others find helpful for their safety performance development.	3.99	0.843
SI2IQ2: I coach and mentor others about safety.	4.09	0.912
SI2IQ3: I personally invest time and effort in developing others’ safety performance.	3.66	0.940
SI2IQ4: I provide on-going safety mentoring.	3.94	0.880
SI2JQ1: In safety matters, I convince others by getting support from key people.	3.82	0.872
SI2JQ2: In safety matters, I convince others by appealing to their self-interest.	2.91	1.373
SI2JQ3: In safety matters, I convince others by engaging them in discussion.	3.70	0.889
SI2JQ4: I anticipate how others will respond when trying to convince them.	3.75	0.832
SI2KQ1: I lead by building pride in the group.	3.91	0.912
SI2KQ2: I lead by bringing out the best in people.	4.20	0.795
SI2KQ3: I lead by inspiring people and articulating a compelling vision for our safety.	4.00	0.865

**Table A3:** Descriptive items and statistical analysis of cognitive competency (CI) variables.

Variable: Description	Mean	Std. Deviation
CIMQ1: I see a situation as multiple cause-and-effect interactions impacting safety.	3.84	0.836
CIMQ2: I explain how certain things affect others resulting in a particular outcome that may affect safety.	4.01	0.757
CIMQ3: I consider safety when explaining complex processes.	4.08	0.778
CIMQ4: I explain an accident in terms of how multiple factors involved affect each other and consequently affect safety.	4.07	0.831
CIMQ5: I see a safety-related accident as a set of cause-and-effect relationships.	4.11	0.758
CINQ1: I perceive similarities among different types of situations.	3.95	0.785
CINQ2: I identify patterns or trends in seemingly random information.	3.78	0.813
CINQ3: I perceive common trends in work accidents.	3.94	0.854
CINQ4: I use examples or stories to describe themes or patterns in an accident.	4.06	0.914
CINQ5: I interpret a new situation by using a story relating it to a different type of situation.	3.77	0.948
